# Clinical-Genomic Analysis of 1261 Patients with Ehlers–Danlos Syndrome Outlines an Articulo-Autonomic Gene Network (Entome)

**DOI:** 10.3390/cimb46030166

**Published:** 2024-03-19

**Authors:** Golder N. Wilson, Vijay S. Tonk

**Affiliations:** 1 Department of Pediatrics, Texas Tech University Health Sciences Center, Lubbock, TX 79430, USA; 2 KinderGenome Genetics Private Practice, 5347 W Mockingbird, Dallas, TX 75209, USA; 3 Director of Medical Genetics and the Cytogenomic Laboratory, Department of Pediatrics, Texas Tech University Health Sciences Center, Lubbock, TX 79430, USA; vijay.tonk@ttuhsc.edu

**Keywords:** Ehlers–Danlos syndrome (EDS), connective tissue dysplasia, dysautonomia, whole exome sequencing, clinical genomics, collagen genes, mitochondrial DNA

## Abstract

Systematic evaluation of 80 history and 40 history findings diagnosed 1261 patients with Ehlers–Danlos syndrome (EDS) by direct or online interaction, and 60 key findings were selected for their relation to clinical mechanisms and/or management. Genomic testing results in 566 of these patients supported EDS relevance by their differences from those in 82 developmental disability patients and by their association with general rather than type-specific EDS findings. The 437 nuclear and 79 mitochondrial DNA changes included 71 impacting joint matrix (49 *COL5*), 39 bone (30 *COL1/2/9/11*), 22 vessel (12 *COL3/8VWF)*, 43 vessel–heart (17*FBN1*/11*TGFB*/*BR)*, 59 muscle (28 *COL6/12*), 56 neural (16 *SCN9A*/*10A*/*11A*), and 74 autonomic (13 *POLG*/25porphyria related). These genes were distributed over all chromosomes but the Y, a network analogized to an ‘entome’ where DNA change disrupts truncal mechanisms (skin constraint, neuromuscular support, joint vessel flexibility) and produces a mirroring cascade of articular and autonomic symptoms. The implied sequences of genes from nodal proteins to hypermobility to branching tissue laxity or dysautonomia symptoms would be ideal for large language/artificial intelligence analyses.

## 1. Introduction

Analyzing the genetic basis of common, multifactorial traits like hypermobility [1] is now possible with the all-gene or genomic screening made possible by NextGen DNA sequencing technologies [2,3]. Advantaging this genomic approach requires equally broad perspectives on diseases like Ehlers–Danlos syndrome (EDS, [4]), evaluating all of its joint (articular) and neurovascular (autonomic) [5] findings rather than the few highlighted by rare types [6,7,8]. If EDS is recognized in its most common form, affecting a significant portion of the 10 to 20 percent satisfying “double-jointed” criteria [1,9], then more studies will be added to those [10,11,12,13,14] finding multiple gene alterations in EDS patients.

Tying multiple genes to the pleiotropic manifestations of EDS also obeys evolutionary precepts that include (a) the ancient origin of connective tissue as the cement of metazoan transitions [15], (b) the necessary diversity of connecting proteins and their regulators that arose in precursor mesenchyme [16], and (c) the more recent elaboration of skeletal and neurovascular elements to support human upright posture [17]. The first precept explains the early innovation of triple helix collagen with its expansion to 28 types that include our most abundant human protein [18]. The second anticipates the many functions of collagens, acting in immune [19], muscular [20,21], and inflammatory networks [22]. Third and most important for understanding EDS symptoms is the balance of orthostatic stability [23] with brain blood supply [24] that, when impaired, produces parallel findings of tissue dysplasia, skeletal deformation, and dysautonomia [11,14,25,26,27].

These clinical-genomic considerations combine to emphasize that multiple genes must be associated with connective tissue functions, variously encoding elements of encapsulation/flow (heart–vessel), matrix/structure (bone–blood–clotting), boundary/constraint (skin–muscle), activity/locomotion (joint–nerve–muscle), and autonomic regulation (circulation–immunity–inflammation). It is then expected that the genes altered in EDS will be accordingly diverse, with 317 of them suggested to participate in networks that include genes predisposing to COVID-19 severity and persistence [14]. Here, the hypothesis of polygenic/multifactorial contribution to EDS is examined by mapping these mutated genes to define their nuclear and mitochondrial chromosome locations, contrasting them with those found in patients with developmental disability, and then correlating their impacts on connective tissue elements with their effects on EDS finding profiles.

## 2. Materials and Methods

A prior report [14] describes how the 1979 EDS patients were evaluated (Supplementary Materials Table S1) and how their DNA testing was coordinated (Tables S2–S4). Only methods relevant to the current data are repeated or expanded here.

### 2.1. Patient Evaluations

Evaluations of 1979 EDS (1899 diagnosed, 1261 systematically evaluated) and 735 developmental disability patients (expanded from reference [27]) were conducted from July 2011 through October 2020 (Table 1). The sole focus on EDS began in August 2017, shifting to online/telemedicine interaction in June 2018. Systematic evaluation of 120 history and physical findings found in the first 915 EDS patients was performed on three groups of EDS patients: 741 seen in clinic, 277 seen in clinic with retrospective form completion (many with salient DNA findings seen before the 120 finding forms were adopted), and 243 evaluated by online/telemedicine interaction where patients filled out the forms. Patients were evaluated and tested in the Dallas private practice of author G.N.W., and their results were collaboratively interpreted and collated with co-author V.S.T. in Lubbock.

### 2.2. DNA Testing

DNA testing of 967 EDS and 461 developmental disability patients (Table 1) used standard methods for whole exome sequencing [2,3] with independent [28] or conjoint [29] microarray analysis. All but 5 EDS patients were tested through the GeneDx Company Gaithersburg MD USA, their requisitions having consented to the anonymous sharing of DNA results, as did clinic intake forms that included consent for medical genetic evaluation/treatment.

### 2.3. Patient and DNA Databases

The 1979 EDS and 735 developmental disability patients having outpatient evaluations were entered into a password-protected MS Excel^©^ GW patient database as described [11,14,27], approved by the North Texas IRB (centered at Medical City Hospital, Dallas, TX, USA) in 2014 (exempt protocol number 2014-054). The 1261 EDS patients with systematic evaluations were transferred to a deidentified EDS1261GW1-23 database that included only sex and age range demographics, type of evaluation, source of referral, detailed history–physical findings, and positive/negative but not specific DNA results. This database provides the source data for this article and is in Sheets S5 and S6 of the Supplementary Materials. For additional identity protection, database patient numbers are scrambled compared to those of patients 1–568 with DNA variants in Table S3. Qualified researchers interested in matching DNA and clinical findings can contact author GNW at golder.wilson@ttuhsc.edu for the connecting code.

### 2.4. Classification of Gene Products, Impacts on Tissue Elements and Processes

As before [11,14,27], information on altered genes and their associated disease is provided through (M) numbers that link to Online Mendelian Inheritance in Man (www.omim.org, information accessed from June 2021 to January 2023) in Table S2. Condensed lists of symptoms are provided for the EDS-related diseases in Tables S2 and S3 but not for the less relevant diseases in Table S4 since developmental–intellectual disability is their only discriminating symptom.

### 2.5. Emendation of Finding Frequency Data from Differently Ascertained Patient Groups

Recall bias yielded lower frequencies for many of the 277 retrospective EDS patients mentioned above while patient overcalling led to higher frequencies for the 243 evaluated by telemedicine–online interaction. Correction of these frequencies was necessary in order to add data from the latter patients to those from the more reliably evaluated 741 EDS clinic patients for the purpose of gene group comparisons (e.g., asking if the finding profiles of EDS patients with collagen type V gene changes were different from those with collagen type I DNA variants). Corrections were also required to include male and childhood EDS patients in these gene group comparisons, the latter having sufficient findings for EDS diagnosis but not for an equitable comparison with adults.

The corrective factors shown in Table S1 were derived from the increased numbers of findings in all EDS patients with age [27] and atypical finding frequencies in the few patients under age 10 (9.4 years), excluding them from gene group comparisons. Dividing the finding frequencies of the EDS 1027 females by those of the 169 males over that age (Table S1B, columns F–G) gave ratios to correct the generally lower finding frequencies for males to those expected for females. Because finding frequencies were quite similar for 292 female EDS patients aged 21–40 having clinic evaluations when they were divided into 5-year groups it could be assumed that all EDS females of that age should have identical finding frequencies. Dividing frequencies of these 292 females by those for the 93 retrospective or 122 online EDS females of that age generated ratios to correct the latter patient frequencies to those expected from clinic evaluation (Table S1). These corrective factors allowed the comparison of 31 EDS patient groups, 516 total patients with gene changes including 448 females and 68 males, 247 having clinic, 179 retrospective, and 90 online evaluations (Table S1B).

### 2.6. Statistics

Clinical findings were tallied from the EDS1261GW1-23 database (Sheets S5 and S6 of the Supplementary Materials), gene and DNA variants from the data in Tables S2–S4 using the search, find, and sort functions of MS Excel©. Statistical averages, standard deviations, standard errors, and coefficients of variation were calculated using its formulae. Significant differences at the *p* < 0.05 level were determined using standard formulae and online resources [30], the latter comparing means by two-tailed t and proportions by N-1 chi-squared tests.

## 3. Results

### 3.1. Different Implications of DNA Variants in EDS and Developmental Disability Patients

Results from DNA testing of 967 EDS patients were documented in the prior article [14] for the purpose of comparison with those influencing the severity of COVID-19 infection. Here, the genomic distribution and potential roles of these variant genes in EDS pathogenesis are examined in more detail, a prime concern being the establishment of a contributive rather than coincidental relation of these gene variations to the EDS diagnosis. A major argument for relevance involves the differences between DNA variants found in EDS versus developmental disability (DD) patients as detailed in Tables S2–S4 and summarized in Table 1.

Complicating the latter enterprise are the significantly higher numbers of female EDS patients with DNA variants (480 of 568 or 85%, Table S3) than 40 of 82 or 49% of DD patients (Table S4) and the fact that whole exome sequencing was usually performed in disability patients after microarray analysis [28] was normal. Also different are the 44 relatives of 18 EDS index cases among the 568 who had positive DNA results, with no relatives being among the 82 DD patients.

### 3.2. Defining the Genetic Basis of EDS Requires Clinical Qualification of Its DNA Variation 

A novel clinical protocol ([14], reprinted below in Table S3 for convenience) converted nominal consensus qualification of DNA variants to ordinal 0–4+ medical diagnostic utility scores. The first steps [DEFG] added Ramachandran conformational assessment of product disruption (D) [31] to the usual evolutionary (E)/functional (F) and gene–disease association (G) [32,33]) considerations of consensus guidelines [34,35]. The second variant impact (Vi) column applies usual consensus descriptors of pathogenic versus uncertain significance, but as a first rather than last step of variant qualification (variants qualified as benign constitute most of the ~12,000 DNA changes found in the average whole exome sequencing analysis [36] and are not reported by commercial laboratories).

The next GHI step of the protocol re-examines the linkage of variant genes to disease, focusing on underlying disease mechanisms rather than specific signs or symptoms. This clinical approach is exemplified by the qualification of *POLG* and *FLG* gene variants discussed below. Relevance is defined by prior associations of gene variants with diseases (e.g., in Table S2) and is diagrammed in the protocol as a dynamic relationship that increases or decreases as (1) DNA testing results accumulate, (2) disease mechanisms become better defined, and (3) the actions of disruptive variants are found in concert or conflict with these mechanisms. Complementing favorable G (gene–disease relevance) scoring can be (4) a more definitive history (H) of disease symptoms and (5) inheritance/concordance (I) of the same variant and symptoms in one or more family members.

These clinical correlations culminate in the penultimate qualification of the protocol, each of the primary DNA variants in 568 EDS and 82 DD patients assigned ascending degrees of diagnostic utility (V*DU), the asterisk indicating no (VnoDU), uncertain (VUDU), moderate (VMDU), strong (VSDU), or evidenced (VEDU) diagnostic utility (Table 1, Tables S3 and S4). Finally, the protocol adjusts (J) qualification for additional variants, those reported as possibly significant but judged less relevant to tissue laxity-dysautonomia or disability pathogenesis than the primary variant. The 327 additional variants in EDS and 68 in DD patients (Table 1) were similarly qualified by their diagnostic utility but also by whether their disease associations (Table S2) supported synergistic (V*DUS) or other (V*DUO) actions [37] of their variant genes. The presence of additional variants judged to have moderate, strong, or evidenced synergism with the primary DNA variant added a plus to the final medical diagnostic utility score (MDna 0–4+, Tables S3 and S4). 

The importance of qualification based on disease mechanism is shown by the qualification of DNA sequence variants in the mitochondrial polymerase gamma (*POLG*) gene [38,39]. Association of that gene with gastrointestinal/autonomic disease (M613662+, Table S2) qualifying the *POLG* variants of patients 116 and 460–473 of Table S3 with diagnostic utility for EDS, while its alternative association with neuromuscular (M607459+) diseases related the *POLG* variants of patients 60 and 82 in Table S4 to their developmental disability. A similar approach to variants in the profilaggrin (FLG) gene recognized its association with immunity and inflammation [40] and related those variants to the mast cell/skin laxity mechanisms of EDS, not to the single finding of scaly skin (M146700) as carried out by commercial laboratories (see later).

### 3.3. EDS/Developmental Disability Differences in DNA Testing Results

Clinical correlation with these broader findings of EDS (Table S1) qualified the DNA variants or variant combinations in 566 or 99.6% of the 568 EDS patients as relevant to their disease profile as opposed to 20 or 3.5% so qualified by the commercial laboratories (left upper column, Table 1). The laboratories were more accepting of DNA changes related to developmental disability, qualifying 48 of 82 (59%) DNA variants as likely or definitely pathogenic (right upper column, Table 1). The stepwise qualification protocol below Table S3 qualified only 1 primary and 14 additional variants in EDS patients as the unhelpful variant of uncertain diagnostic utility (VUDU, VUDUS, middle rows of Table 1) compared to 367 primary variants qualified as variants of uncertain significance (VUS) by the DNA testing laboratories. The laboratories qualified another 181 primary DNA variants as pathogenic for other diseases since correlation with tissue laxity, neuromuscular, or autonomic mechanisms [11,14] was not recognized.

Reflex whole exome sequencing after normal microarray analysis [28] in most of the disability patients explains why only 112 or 15% of them had this testing compared to 906 or 48% of the EDS patients (legend to Table 1). Of the 459 disability patients having microarray analysis, 102 (22%) had potentially significant copy number variants including 11 of the 76 with positive whole exome sequencing shown in the last column, Table 1 (microarray data not shown). In contrast, only 9 EDS patients had copy number variants found by simultaneous testing [29], with 3 judged relevant to EDS (Table S3). One of the 6 not related to EDS (patient 567 of Table S3) had a 15q13 microdeletion that may have been relevant since the deleted region included the *CHRNA* (M100690) cholinergic receptor gene (M100690). 

Table 1 (middle rows) shows the respective 327 or 68 additional DNA variants in EDS or DD patients that are often ignored in published work, 96% or 44% of them judged to have moderate to evidenced synergistic contribution (V*DUS) to the patients’ diagnoses [14,37]. More (28 or 41%) of the 68 additional variants in disability patients were associated with other diseases (V*DUO) compared to 13 or 2.0% of 911 in EDS patients (Tables S3 and S4). Of EDS patients, 221 or 39% had additional variants compared to 41 or 50% of disability patients. The latter total does not count the 12 chromosome or copy number variants in DD patients that may contribute to their disability. The 17q21.31 microdeletion in patient 33 of Table S4 may have contributed more to disability than its accompanying *G3BP1* (M608431) gene sequence alteration but was rated secondary so that it would parallel the classification of EDS variants. Supporting the idea of an EDS gene network are the similar proportions of patients with three or more variants in EDS (36%) and DD (46%) patients (Table 1 legend) given the many genes associated with intellectual disability [41].

### 3.4. Comparison of Altered Genes in EDS and DD Patients

The 330 gene variants in EDS patients and their prior disease associations are listed in Table S2, with 10 genes with 13 DNA variants not considered relevant to EDS at the bottom, along with 3 genes and 5 variants considered incidental or secondary findings [42]. The 917 DNA variants in 568 EDS patients are listed in Table S3 by patient number. Single and therefore primary variants have 0.0 after the patient number, multiple variants followed by 0.1 for the one judged primary, and 0.2, 0.3, etc., for additional variants. When two or more variants occur in the same gene, they are given separate numbers and labeled as homozygous (18 variants, 9 patients), trans (47 variants, 23 patients), cis (23 variants, 12 patients), cis-trans (10 patients) or cis + trans (patient 231 with 3 variants) in column E of Table S3. Of the 911 DNA sequence variants cited in commercial reports, 561 (62%) were listed in ClinVar [32] and 71 of 158 mitochondrial DNA variants (45%) were listed in MitoMap ([33], see Table S3 column D and legend).

Patients are numbered from low to high according to how much their altered genes are thought to contribute to EDS, those with variants in well-recognized genes like collagen type V ([6,43,44,45,46,47,48,49,50,51,52,53] variants) having low numbers and those given novel relevance by this study (e.g., collagen type VI—12 variants, or mitochondrial ATP synthase—32 variants having higher numbers). The disability variant list in Table S4 is similarly numbered and qualified but ordered by date of entry since all were relevant to developmental-intellectual disability.

A striking number (143 or 45% of the 317 EDS-relevant genes in Table S2) would satisfy consensus guidelines for causality, their central to peripheral positions in the postulated EDS–dysautonomia gene network indicated by their numbers of relevant variants. The 14 genes with 10–40 variants in EDS patients (Table S2) would meet the *strong* evidence criterion for EDS gene association by MacArthur et al. [35], the 71 genes with 3 to 9 variants in EDS patients their *moderate* evidence criterion, the 58 genes with 2 EDS-related variants and the 174 with one relevant variant needing additional EDS patient variant observations for validation. Important for these EDS gene correlations is the realization that mutations in different regions of these genes can cause different patient symptoms [38] and that alterations in different genes can combine to cause disease by acting in a network fashion [14,41,44].

A recent study [13] also found many of these same variants in EDS patients, several previously associated with other diseases (e.g., in the *TGFB2/3* genes associated with Loeys–Dietz syndromes (M614816+) and the *COL6/12* genes associated with Bethlem myopathies (M158810+)). As with many of the variants in Table S3, several of their heterozygous variants were in genes associated with recessively inherited disorders (e.g., *ITGB3* with blood diseases (M616913), *ZNF469* with brittle cornea syndrome (M229700)).

There were 20 genes (with 24 variants in 21 disability patients) that were also variants in EDS patients (blue colors in Tables S3 and S4). Nuclear genes include *ATP7A*, *DUOX2*, *FLNA*, *POLG*, and *TG*, mitochondrial ones include *MT-CO2*, *MT-TK*, and *MT-ND5*, and their different mutations feasibly contribute to cognitive disability on the one hand or to the autonomic and neurologic issues of EDS on the other. Also in both patient groups were the profilaggrin gene *(FLG*, M135940) variants, present in 2 (2.4%) of the 82 disability and 35 (6.2%) of the 568 EDS patients (Tables S2–S4). Only in the latter group was their prevalence more than 2.2% in normal individuals [40], supporting their autonomic–inflammatory and/or skin fragility effects in some EDS patients. Additional variants in the connective tissue-related *COL11A*, *PLOD1*, and *FBN2* genes in disability patients may augment the hypermobility that results from CNS-related hypotonia as reported in a child with Down syndrome [45].

### 3.5. Differences in Variant Origin

**Figure 1:**
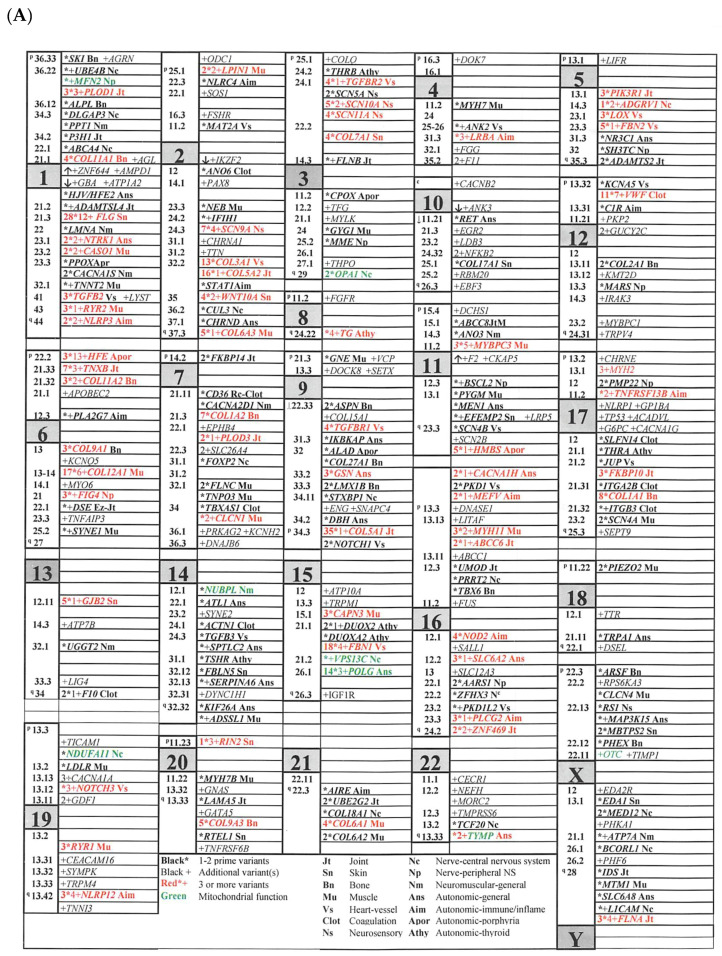

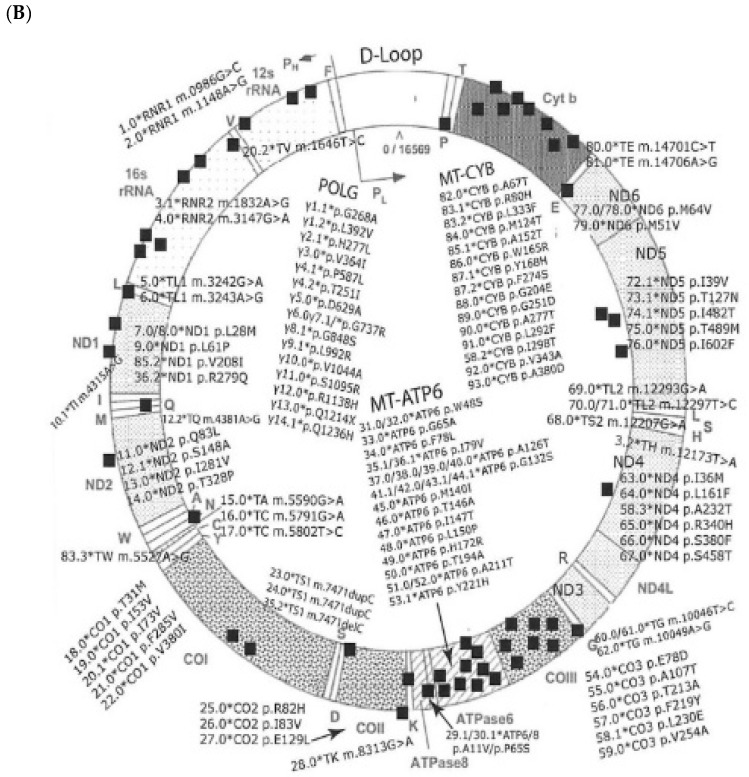
Maps of nuclear and mitochondrial genes with variants in EDS patients. (**A**) Nuclear genes from Table S2 are shown with numbers of primary variants in bold followed by *, of additional variants in italics followed by +, genes with 3 or more variants in red, genes encoding products transported to mitochondria in green; gene abbreviations and exact loci are in the Table S2; chromosome sizes are modified for display by factors ≅x1/2 for numbers 4-5-9, x1/4 for 8, x2/3 for 10; x1.1 for 14-21-X; x1.3 for 22, x1.7 for 20; x2 for 16-17-19 [25]. (**B**) Primary DNA variants are described by DNA (m.) or protein (p.) position, additional ones positioned by ■—see variant details in Tables S3 and S4. The nuclear gene *POLG* is listed because of its importance in mitochondrial replication, all others are variants of mitochondrial DNA; the Figure 1B map is from MITOMAP [33].

The lower rows of Table 1 show statistically significant differences in the origins of DNA variants in EDS versus disability patients, primary nuclear variants having maternal origin in a statistically significant 35% versus 18%, while de novo variants have the reverse difference of 3.8 versus 48%. Another difference from disability patients is the prevalence of mitochondrial variants in EDS patients (158 versus 10, bottom of Table 1, mapped in Figure 1B), surprising in view of their associations with severe disability diseases such as Leigh syndrome (M256000). The frequency of mitochondrial variants and nuclear variants of maternal origin correlates with the 18 (sons) to 22% (daughters) transmission rates of EDS from affected mothers, the 4.4 (sons) to 2.7% (daughters) rates from affected fathers, and the presence of affected mothers in an average 59% of affected EDS patients compared to an average 23% who had affected fathers [11].

### 3.6. An EDS Gene Network Spread over Multiple Chromosomes Including That of Mitochondria

The 65 genes with 3 or more variants in EDS patients (moderate to strong causality [35]) have a broad distribution in the nuclear genome (Figure 1A—bold, red print), matched by 30 of 37 altered genes in the mitochondrial genome of Figure 1B, their primary DNA variants having specified DNA/protein changes. With less certain relevance but equally wide distribution are the 252 genes with fewer than 3 variants (bold, black print in Figure 1A), 110 of them with no primary and only additional variants (italic, black print in Figure 1A, filled black squares in Figure 1B). Primary variants are indicated by * symbols in Figure 1A.

Genes are classified by their impact on tissue elements (e.g., joint, Jt) or processes (e.g., Ans, general autonomic regulation) according to their previous associations with disease, as shown in the lower box of Figure 1A and the legend of Table S2; these classifications are listed beside the genes with primary variants. Variants in nuclear genes that encode products routed to the mitochondrion are in green print in Figure 1A and listed for the nuclear gene encoding mitochondrial DNA polymerase gamma (*POLG*) in Figure 1B. The 17 POLG variants with their linkage to neuromuscular (M607459+) and dysautonomia (M612662+) symptoms [38,39], echoed by the diverse mitochondrial DNA variants of Figure 1B, suggest mitochondrial depletion with brain–muscle energy deficiency as the way in which mitochondrial dysfunction contributes to EDS. EDS-associated variants in the *OPA1* dynamin-like GTPase and *TYMP* thymidine phosphorylase nuclear genes, associated, respectively, with mitochondrial depletion diseases M616896+ and M603041, support this suggestion.

Another classification in Table S2 important for later comparisons pertains to the nature of the RNA or protein product encoded by the gene, terms like Ez for enzyme, Mc for membrane channel, or Tf for transcription factor, etc., explained in that Table legend. The transcription factor group includes 26 or 8.2% of the 317 genes relevant to EDS ([14], Table S2) and suggests that many EDS-relevant mutations in regulatory regions outside of exon or exon–intron borders remain to be discovered. The diverse element-process impacts and products of EDS genes are paralleled by their diffuse genomic locations, clustering evident only for *COL5A2/COL3* at 2q32.2, *SCN5/10/11A* at 3p24.1, *COL6A1/A2* at 21q22.3, and *SCN2B/4B* at 11q23.3.

### 3.7. Holistic Evaluation and Quantification of EDS Findings Allows Comparison of Patients with Different Gene Changes 

The 80 history and 40 physical findings listed in Table S1A [11,27] were designed to optimize EDS diagnosis by including 20 consensus criteria [46] for hypermobile [1] and 16 for classical [6] EDS, as shown by the bolded h or c letters beside the findings (criteria for these EDS types are to the right of Table S1B, columns AU-AV). The findings were listed in 12 history (Hx) and 7 physical (PE) categories (boxed in Table S1A), the total number of history or physical findings (rows 5–6 of Table S1A) providing numerical criteria for EDS diagnosis (see Table 2 below). Category totals measure the prominence of EDS characteristics (e.g., hypermobility by the number of Beighton maneuvers performed—row 7) or the severity of its complications (e.g., joint instability injury by the number of joint findings—row 15). 

The systematic evaluation outlined in Table S1A includes traditionally emphasized EDS findings [1,4,5,6,7,46] related to joint laxity (subluxations row 17, aware of flexibility row 105), joint injury (fractures row 23, early joint pain row 107), skeletal bends or deformations (scoliosis by history row 27 and physical row 35), skin fragility (unusual scars by history row 43 or physical row 52), and rarer cardiovascular findings (aneurysm, row 123). Underappreciated consequences of autonomic imbalance include findings of postural orthostatic tachycardia (POTS [47,48]—chronic fatigue row 56), mast cell activation (MCAS [49,50]—migratory rashes row 73), and irritable bowel syndromes (IBS [51]—irregularity row 67). Contrary to prevailing opinion [6,46], these findings of adrenergic excess (POTS, MCAS) and cholinergic suppression (IBS) will inevitably accompany connective tissue laxity and vessel laxity because the circulation of dependent blood to the brain relies on sympathetic stimulation [11,14,25,27].

Neuromuscular findings [52] like numbness–tingling and neuropathy (rows 58 and 87) or poor balance by history (row 86) or physical (row 95) are also included to emphasize the cycling from vessel laxity to autonomic imbalance that, through its small fiber (autonomic) neuropathy [53], reciprocally enhances tissue laxity. This range of findings and category totals thus delves beneath superficial diagnoses like fibromyalgia [54] to profile most of the systemic, mechanistic, and age-related manifestations of EDS–dysautonomia [14].

Pertinent here are comparisons of these finding frequencies and category totals among EDS patient groups: first, among those with and without EDS to validate their diagnostic utility, second, to quantify differences between EDS patients of different sex or method of evaluation (clinic, retrospective, online—see Methods), and third, to compare finding profiles in EDS groups with particular gene changes after adjusting for sex and ascertainment differences. Table 2 shows the first two comparisons and begins the third by outlining the profiles of EDS patients with nuclear, mitochondrial, and collagen type V gene changes.

Table 2 (and the later Table 3) select and combine certain finding categories from Table S1A to show their average totals, e.g., the number of history (out of 80) or joint-skeletal history plus physical (JtSktH + P) findings (out of 21) in data rows 5 and 10. These Tables also compare the average frequencies of findings classified by their underlying mechanism from Table S1B: 50 of the 120 in Table S1A with higher frequencies were selected and placed in 10 mechanism classes (another 10 findings with bearing on management are also compared among gene groups. Among the 10 were 8 classes of 5 findings caused by different aspects of the same joint hypermobility, skeletal deformation, dysautonomia-POTS, or dysautonomia–IBS/MCAS mechanism; these were combined to make 4 classes of 10 that, when combined with skin fragility and neuromuscular mechanism findings, made 6 total (Table 2 legend). Thus, category totals alternate with mechanism-related finding frequencies in Table 2, the JtSkH+P total of row 10 beneath the DF axial–limb deformations average in row 9.

### 3.8. Comparison of Quantified Tissue Laxity/Neuro-Autonomic Findings in EDS Patient Groups

The first three columns of Table 2 reinforce the previously demonstrated [27] differences between patients diagnosed with EDS (37/19 history/physical findings in EDS females, 27/17 in EDS males) and those not meeting EDS criteria ([46]—7.4/7.7). Exceptions include the average number of Beighton maneuvers [9] performed by the male EDS and Not EDS patients (5.6 of 9) in Table 2 and certain finding frequencies in Table S1B columns F-H, like the 46/43% (row 51) of EDS males/Not EDS patients having subluxations, the 45/61% having early joint pain (row 55), and the 39/30% having colic-infantile feeding problems (row 115). Their joint hypermobility/instability with early pain and feeding issues [25] explains why many of the Not EDS patients were referred for that diagnosis but did not have sufficient history–physical findings (over 10 of each, [27]) to receive it. Although their exclusion validates the systematic evaluation as a diagnostic tool, the higher percentage of males in the Not EDS group (28 or 50% in Table S1B compared to 169 or 14% of all EDS patients in Table 2) and their younger age (averaging 15 years versus 31/23 for EDS females/males in Table 2) indicate that they are not a true control group.

Noted that 17 of the 20 consensus findings of hypermobile and all 16 of them for classical EDS were included in the category totals and mechanism-related finding percentages shown in Table 2, Table 3, and Table S1. This means that the 1261 patients with systematic evaluations including the 568 with positive DNA testing results would definitely meet consensus diagnostic criteria for EDS [4,46]. The second row of Table 2 shows that 69% of EDS females and 75% of EDS males met the 2017 criteria for the hypermobile type of EDS ([1,46], right of Table S1B). This diagnosis reflected more joint laxity (subluxations, Beighton maneuvers) and typical skin findings (flat, white, atrophic versus raised, discolored, keloid-like scars) than seen with classical EDS [6]. Comparable hypermobile EDS majorities were found in all Table S1B groups save those with COL5A1 DNA variants, refuting the evolutionarily implausible assumption [1,4] that patients with this type had no gene changes.

The previously demonstrated severity of EDS in females [27] compared to males is again shown by their significantly higher category totals in Table 2 for all but their age of onset. Significantly higher finding class frequencies are also shown for all but the deformation mechanism (42 versus 43% in row 9 of Table 2), reflecting higher or near-equal male frequencies for the axial deformations of tall stature, long face, high palate (rows 62–64) and all of the limb deformations like long fingers or flat feet (rows 68–72). Less muscle constraint and the need for pelvic expansion during parturition are two reasons for the intrinsically greater flexibility of females [27] and male frequencies corrected as discussed in Methods, so EDS gene groups with different sex ratios could be compared.

The next three columns of Table 2 show similar frequencies for all of the EDS patients ascertained in the clinic, retrospectively, or online. The latter two patient groups still show minor differences from the more reliably evaluated clinic patients after corrections based on comparably evaluated 21–40 year females, as indicated in Methods (corrective factors shown in red, Table S1B). The large number of patients over 10 (≥9.5 years) in these groups gives statistical significance to the total history, Beighton score, joint-skeletal, and skin-finding differences of the retrospective and online groups (Table 2). Only 6 of the 62 findings (jaw skin stretch, asthma, muscle aches, poor balance, motor delay, heart defects) show significant differences in the retrospectively assessed patients and only 1 (dysphagia) in the online group (columns EFG, rows 26–44 of Table S1B).

The similar finding profiles of 437 EDS patients with nuclear genes and 79 with mitochondrial DNA changes in Table 2 (data columns 7–8) preface the similarity of patients with their individual genes in Table 3. Only the category totals of IBS-MCAS and neuromuscular (NmH + P) findings are significantly lower for patients with mitochondrial DNA change (lower rows of Table 2). These differences and those of patients with individual mitochondrial gene changes (*MT-ATP6*, etc.) in Table 3 suggest that the contribution of mitochondrial dysfunction to EDS is similar to its role in aging [55] and different from its involvement in severe neurologic disorders [38].

Also foreshadowing similarity of all gene groups are those with collagen type V gene changes (Table 2, last three columns). That 53 EDS patients had *COL5A1* and *COL5A2* DNA variations (Table S2) that have long been associated with EDS [1,6], 49 of them old enough for clinical comparison, gives additional support for EDS relevance of the 566 DNA changes so designated in Table 1. These numerous patients with accepted gene changes not only provide a reference for others in Table 3 but also one opportunity to compare the clinical consequences of genes encoding different peptide chains of a collagen triple helix.

The one significant difference in Table 2 was a higher percentage of patients meeting the criteria for hypermobile EDS in those having *COL5A2* gene change, fitting with the association of *COL5A1* gene changes with classical EDS (M130000, classical EDS type 1) but not with the similar association of *COL5A2* gene changes (M130010, classical EDS type 2). The latter gene’s association with hypermobile EDS is further supported when the individual finding frequencies of Table S1B (columns O-P, rows 11–13) are inspected: *COL5A2* patients have significantly more child clumsiness and awareness of hypermobility, of Beighton maneuver and reverse prayer performance, in the HI-joint-flex class of findings, than the *COL5A1* patients. Their higher percentages of many neuro-autonomic findings (rows 28–42) correlate with their greater flexibility and the fact that the *COL5A2* gene contributes a pivotal single chain to the type V triple helix [43,56].

### 3.9. An EDS Gene Network—Recurring Gene Variants Produce Congruent Clinical Profiles

EDS patient groups with multiple variants in the same gene and their finding frequencies are detailed in Table S1B and summarized in Table 3. The gene names and M number references with associated diseases are listed in Table S2, along with classifications of their product type (e.g., enzyme or transcription factor), targeted tissue (e.g., joint or muscle), or process (e.g., general autonomic—Ans, autonomic–immune–inflammatory—Aim, neuromuscular transmission and contraction—Nm). These product types and tissues/processes impacted are listed in the lower box of Figure 1A and the Table S2 legend. The tissue process impact was determined by the symptoms of associated diseases, sometimes arbitrary when genes were associated with pleiotropic syndromes and/or multiple diseases (M+ symbol).

- Genes associated with impact on joints, bone, and skin produce similar EDS profiles

Note the many patients with changes in genes that were formerly associated with particular types of EDS or related disorders who have typical EDS finding profiles (Table 3A). There are over 30 such genes in addition to *COL5*—(a) 22 with heterozygous variants in affecting genes (COLenzyme) encoding collagen-processing enzymes (COL enzymes) like *PLOD1* q. v. recessive kyphoscoliotic EDS (M225400—[8]); (b) 14 with *COL1* variants q. v. osteogenesis imperfecta or EDS (M166200 or M619115—[19,57,58]); (c) 16 with *COL2-9-11* variants q. v. Stickler syndromes (M108300, M614134); and (d) 15 with clotting/*VWF* variants q. v. von Willebrand disease (M143900, [59]), and other genes sharing VWF domains [22,60]. The emphasis on the skin by Ehlers and Danlos is fulfilled by (e) 14 patients with variants in skin-impacting genes (including 6 in both *GJB2* associated with keratitis, M148210 and *WNT10A* associated with ectodermal dysplasia, M2570980+); (f) the 5 patients with *COL7* or *COL17* variants [61] associated with blistering skin diseases (M131750, M619787); and (g) the many (25 patients) with mutations in the aforementioned profilaggrin (*FLG*) gene with its scaly skin (M146700) and eczema (M605803) associations.

The group with collagen processing alterations (COLenzyme) shows few differences in joint-skeletal or other features in Table 3A, re-emphasizing that heterozygous mutations can produce typical EDS findings when operating in networks (44) and that even biallelic variants (as in patient 58 of Table S3 with PLOD3 lysyl hydroxylase-3 variants, M603066) will not necessarily produce a specific type of EDS. The *COL1* group has significantly more joint-skeletal problems (averaging 11 in Table 3A versus 9.4 for all 516 EDS patients) and notably more fractures (79% versus 51% in Table S1B), as expected from their prior association with brittle bone diseases (M166200). More MCAS complications occurred in the COL2/9/11 patients (5.6 compared to 4.6 for all in Table 3A), with higher frequencies of rashes and asthma suggesting that the arthritis associated with Stickler syndrome (M120140) may have inflammatory as well as wear-and-tear causes.

Patients with alterations of VWF and other genes associated with clotting also have typical EDS profiles in Table 3A, the more bruising, striae, and pedal blood pooling in Table S1B signs of vessel fragility and distensibility expected from alterations of a gene that can produce the von Willebrand pattern of nose and postoperative bleeding. The EDS profile of this group also indicates that the connective tissue important for clotting and vessel wall adhesion has general roles in the skeleton and skin. The groups with other skin and C*OL7/17* variants show the important role of skin constraint in maintaining connective tissue, having, respectively, more physical findings (averaging 21 versus 19.5 for all) and joint-skeletal findings (12 versus 9.4 for all) in Table 3A. The other skin group had higher frequencies of soft skin, easy bruising, and unusual scarring in Table S1B, though the COL7/17 group did not, a difference shared by the *FLG* group [40] with more history (41 versus 38) and IBS-MCAS findings (5.5 versus 5.1), shown in Table 3A. The latter group also showed a general increase in dysautonomia findings such as syncope, chronic fatigue, colic-feeding problems with later weight loss, and thyroid/heart changes in Table S1B. As discussed above, these FLG mutations that may produce the common finding of scaly dry skin in most people (2.2% prevalence in several cases, Table S3) have as much impact on inflammation as on skin integrity [62]—the reason that they are classified as affecting autonomic–immune–inflammatory (Aim) processes in Table 3A, Tables S1B and S2.

- Genes associated with impact on heart and vessels

Congruent finding profiles continue with genes impacting the heart and vessels, notably the 12 qualifying patients with *COL3* variants q. v. vascular EDS (130050 [7,63]), the 17 with *FBN1* variants q. v. Marfan (M154700+, [64]) and other disorders, the 11 with transforming growth factor/receptor genes q. v. Loeys–Dietz (M609192+ [65,66,67]) syndromes. The latter patients’ compatibility with EDS is reaffirmed by the exclusion of patients with the obvious clinical diagnoses of Marfan or Loeys–Dietz syndromes from this study. The *COL3* and *FBN1* patient groups do have higher numbers of findings in most categories and significantly higher frequencies of several including for IBS-MCAS findings (lower rows of Table S1B). More findings comport with genes associated with severe diseases [7,64] and pose the question of whether TGF pathway medications like Losartan will be useful for EDS patients [68]. Continuing the theme of clinical congruence with subtle differences in certain findings (Table S1B) are higher frequencies of long fingers and tall stature in *FBN1* patients, of several POTS symptoms in all the heart–vessel (Vs) patients, and of bowel irregularity–bloating–dysphagia in *COL3* patients whose vascular EDS homologs have high risks for bowel ischemia [7].

- Nuclear/mitochondrial genes associated with neuromuscular diseases produce similar EDS profiles

The high frequencies of dysautonomia/neuromuscular, as well as the tissue laxity findings in Table S1, predict that alterations in genes associated with neurologic disorders would contribute to EDS. Table 3B in its last columns depicts typical EDS profiles from genes affecting the central (Nc group) and peripheral motor (Np group) or sensory (Ns/SCN9A-11A group) nervous systems. The Np genes that were formerly associated with various forms of Charcot–Marie–Tooth disease (M188200+, [69]) produce significantly more neuromuscular findings as would be expected (7th column, last row). The genes impacting nerve to muscle innervation (Nm group) are allied with the 4 mitochondrial gene groups of Table 3C; the latter convey an abundance of neuromuscular symptoms [70] in their sparsely described disease associations (Table S2, e.g., M516000).

In contrast with these neuromotor associations, the diseases associated with SCN9-11A gene mutations [71] have dysesthesia (e.g., small fiber neuropathy, erythermalgia M615552) or dysautonomia symptoms (hereditary sensory and autonomic neuropathy M615548) that are associated with unmyelinated (autonomic) neurons. Their congruent EDS profiles in Table 3B support the idea that autonomic dysplasia can reinforce articular symptoms. As discussed above, the 13 patients with POLG mutations [38,39] relate to two types of disorders—a neuro-autonomic disorder (M613662+) or a mitochondrial depletion syndrome (M203700+)—while the 18 with MT-ATP gene changes [72,73] relate to a disease with neuromuscular and dysautonomia symptoms (M516060). These and the many other mitochondrial mutations in EDS patients (Table 1, Figure 1B) further demonstrate the ability of mitochondrial dysfunction to influence articulo-autonomic dysplasia.

Note that 61 patients have changes in genes encoding components of all 4 respiratory complexes of the mitochondrion [74] in Table 3C, each group having a similar EDS–dysautonomia profile of findings. These finding patterns are also similar to those of the 35 patients with muscle-impacting genes (Mu group) and the 24 with COL6-12 mutations [21,45,75,76] q. v. Bethlem myopathies (e.g., M158810), again emphasizing how important fleshy (skin/muscle) support and constraint are for connective tissue integrity [77]. The challenge becomes how to boost mitochondrial and muscle function by exercise that allows joint preservation [20,56,78].

### 3.10. Correlation of Gene Action with Finding Profiles

Table 4 provides additional support for the relevance of these gene variants to EDS and for the idea of tissue laxity or autonomic changes fueling a common cascade of findings that are consequences of an articulo-autonomic cycle. Column 1 divides the 516 EDS patients with gene changes into three groups according to whether their gene actions reflect tissue laxity, dysautonomia, or neuromuscular processes; all of these patients are grouped in its bottom row. Columns 2–3 show the findings tallied for these four groups from the summary rows 11–47 of Table S1B, separated by their relation to the same tissue laxity, dysautonomia, or neuromuscular mechanisms. The total numbers of these findings that are increased (columns 4–5) or decreased (columns 6–7) are taken from those rows of Table S1B, where they are indicated, respectively, by green or red print.

Looking first at the increased or decreased finding frequencies for all groups in the bottom rows of columns 4–7, one sees 65% of findings related to tissue laxity *increased* and 35% decreased, a trend expected since hypermobility and joint pain were prominent reasons for EDS evaluation. Proportionate increases in finding frequencies related to dysautonomia (51%) or neuromuscular (40%) mechanisms were less but still substantial, correlating with the more distant relationships of these findings to the primary EDS indicator of tissue laxity. Now look at the correlations of *increased* finding frequencies with the actions of the genes grouped in column 1: genes impacting tissue laxity had more tissue laxity findings showing increases (67%) than decreases (33%) in the top 3 rows, dysautonomia-associated genes in the next 3 rows, and neuromuscular-associated genes in the following 3 having, respectively, more increases in dysautonomia (65%) or neuromuscular (56%) findings.

Thus, each of the three gene groups contributes to findings correlating with their mechanism of action: *COL2/3/5/7/9/11* and other gene changes in the upper tissue laxity group producing more increases (67%) than decreases (33%) of tissue laxity findings; *MT-ATP* and *POLG* changes, among others, in the second group producing more increases (65%) than decreases (35%) of dysautonomia findings; and *COL6/12*, among others, in the third group producing more increases (56%) than decreases (44%) of neuromuscular findings. Even more striking than the directed but still substantial frequencies in the three mechanism groups are the high proportions of dysautonomia (61%) or neuromuscular (71%) finding increases seen with genes producing tissue laxity (top three rows).

The data in Table 4 synthesizes those from prior tables to emphasize that the connective tissue laxity mechanisms [1,4,5,6,7,8] producing EDS are integrally and reciprocally linked to mechanisms of autonomic imbalance [25,26] and neuromuscular dysfunction [53]. Findings relating to all three of these processes must be included in the diagnostic guidelines for EDS and be holistically evaluated when deciding if a clinical profile warrants separation as a distinctive EDS type. Genomic analyses [10,11,12,13,14] must also acknowledge these articulo-autonomic mechanisms if its potential for EDS precision medicine is to be realized.

## 4. Discussion

The limitations of this study are many, including its cross-sectional nature, the different ascertainment of findings that are often subjectively reported, and the qualification of gene changes by inferred mechanism, all lacking the desired rigor of precise molecular medicine. Nevertheless, its holistic documentation of syndrome findings and DNA change with unprecedented numbers of patients sets standards that are essential if genomics is to achieve its potential for the analysis, prevention, and therapy of complex diseases. While single gene–disease relationships revealed by targeted DNA sequencing have yielded insights and powerful therapies for certain rare diseases [68], common multifactorial disorders affecting connective tissue, neurodevelopmental, and other functions must be explored using the polygenic screening made possible by massively parallel sequencing [2,3,10,11,12,13,14]. Critical for the conjunction of myriad and variable disease findings with our equally volatile genome [36] is experienced clinical correlation, a perspective that matches the old tools of comprehensive history–physical with the new ones of genomics and systems biology.

### 4.1. Envisioning an EDS–Dysautonomia (Articulo-Autonomic Dysplasia) Gene Network or Entome

**Figure 2:**
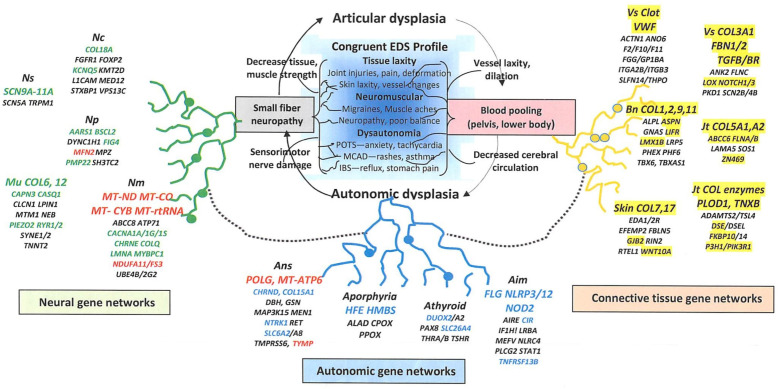
The gene network or entome concept of EDS pathogenesis. Gene variants in EDS patients (Table S3) are grouped by mechanism (Table S2), their interlacing networks impacting reciprocal tissue laxity (articular)-neural (autonomic) processes to produce a reciprocal cascade of EDS–dysautonomia symptoms. Genes with variants in 5 or more EDS patients are in large print, those important for mitochondrial function are in red print, filled circles represent mutations (network nodes), like certain ones in *COL3A1* that overwhelm networks to produce extreme, single-gene disease [7].

The idea of mirroring cascades of genes as roots and symptoms as branches analogous to Tolkien’s Ents was previously expressed as a model that encompassed the overlapping findings of EDS and long COVID-19 [14]. Figure 2 expands the analogy by envisioning the genes discussed in this article as parts of interlaced tissue laxity, dysautonomia, and neuromuscular networks that drive a reciprocally diverse tree of pathogenic mechanisms and symptoms. Three results support a contributive rather than coincidental association of these variant genes to EDS: (1) 51 patients had DNA sequence variations in collagen type V genes that have long been associated with EDS; (2) the 317 genes showing variations in EDS patients were mostly different from the 82 found in those with developmental disability; and (3) many of the genes in Table S2 (orange shading) were previously associated with conditions having over 3 findings of tissue laxity.

### 4.2. Relating Genes to Pathogenic Mechanism Can Guide Clinical-Genetic and Evolutionary Correlation

Current matching of all mutations in a gene with one disorder (e.g., *COL3A1* gene change to vascular EDS M130050), with one disorder type (e.g., *COL5A2* gene change to classical type EDS-2, M130010), or especially with one component sign or symptom (e.g., *FLG* with scaly skin/ichthyosis, M146700) is a reason that so many DNA variations are qualified as the unhelpful variant of uncertain significance. Relating each of these genes to underlying mechanisms (e.g., vessel–tissue laxity or adrenaline-guided inflammation) places them in sequential pathways with like-acting genes that can be correlated with disease patterns rather than with single types or symptoms. Such sequences of gene1–gene2–molecular mechanism–clinical process–symptom1–symptom2 would simulate the word-next-word sequencing of large language models and facilitate analysis by artificial intelligence methods [79].

The genomic perspective and analysis modeled here related an unexpected variety of genes to the processes of articular and autonomic dysplasia in EDS (Table 2, Table 3, and Table S2) and validated the hypothesis of polygenic contribution. Mutated in EDS patients to produce a common EDS–dysautonomia profile were genes previously associated (a) with other connective tissue dysplasias—*ABCC6* [80], *COL1* [58], *COL3A1* [7], *FBN1* [64], *TGFB/BR* [65,66,67], and *ZNF469* [81]; and (b) with other types of EDS including heterozygous variants formerly involved with recessive diseases—*ADAMTS2* [82], *COL5* [43,56], *FLNA* [83], *FKBP14*, and *LOX* [84].

Changes in genes (Table S2) relating to other tissue elements or clinical processes included (c) skin—*COL7/17* [60]; (d) cardiovascular (*SCN2B*/*4B*); (e) clotting (*F10*, *VWF* [59]); (f) central nervous system—*COL18A1*, *L1CAM*; (g) peripheral nerve/Charcot–Marie–Tooth—*AARS*, *PMP22* [69]; (h) sensory nerve—*SCN9A*-*11A* [71]; (i) other neuromuscular actions—*CACNA1A/G/S*; (j) muscle—*COL6*/*12* [74,75], *RYR1*/*2* [85]; (k) autonomic—*COLQ* [86], *HFE* [87], *HMBS*, *POLG* [38], and *SLC6A2*; (l) immune inflammatory—*NLRP12*, *NOD1/2* [88]; and (m) mitochondrial—*MT-ND*/*CO*/*CYB*/*ATP6* [72,73,74,89].

The broad chromosomal distribution without clustering of these nuclear and mitochondrial EDS-associated genes (Figure 1), plus the involvement of many homologous or functionally related genes suggests an evolutionary process analogous to the networks pictured in Figure 2. Homologous genes found variant in EDS patients include *ABCC1*/*6*/*8*, *ADAMTS2*/*TSL4*, *CLCN1*/*4*, *COL2*/*3*/*5*/*6*/*7*/*9*/*11*/*12*/*17*/*18*/*27*, *DSE*/*DSEL*, *EDA1*/*2R*, *FKBP10*/*14*, *FLNA*/*B*/*C*, *MYH2*/*7*/*7B*/*11*, *NLRP1*/*3*/*12*, *PKD1*/*PKD1L2*, *PLOD1*/*3*, *RYR1*/*2*, *SCN2B*/*4A*/*4B*/*5A*/*9A*/*10A*/*11A*, *SYNE1*/*2*, *TGFB2*/*3*, *TGFBR1*/*2*, and *TNFRSF6B*/*13B*, while functionally related genes include *CACNA1A*/*1G/1H*/*1S*, *CHRNA1*/*E*, *F2*/*10*/*11*, *ITGA2B*/*ITGB3*, *SLC6A2/6A8*/*12A3*/*26A4*, and *THRA*/*B.* An operon-like unit encoding a simple collagen bracketed by regulatory sequences likely diversified in early metazoans by duplication and transposition rather than clustered expansion. As novel tissues differentiated, each duplicated unit would develop new domains and additional genes to produce the required connecting and regulating molecules—these common regulators would foster network action.

### 4.3. Holistic Recognition and Relating Findings to Mechanism Can Improve EDS Recognition and Management

A comprehensive and systematic analysis of 120 physical findings in 1261 EDS patients (Table S1A) quantitatively distinguished those meeting EDS criteria who were sufficiently old (over 10 years) to manifest consistent findings [27]. Integral relationships between tissue laxity and neuro-autonomic mechanisms are suggested by their parallel impacts on EDS symptom frequencies (Table S1A, EDS1261GW1-23 database of Sheet 6) and by the congruent finding patterns in EDS patients with changes in genes impacting these different tissue laxity or neuro-autonomic mechanisms (Table 2 and Table 3 and Table S1B). Their parallel actions are also suggested by the overall finding of frequency changes in Table 4.

These results presage an anticipatory approach to the 10–20% of adolescents–adults with hypermobility that (a) screens for joint pain, skin elasticity, activity limitations, urogenital problems, and symptoms of autonomic imbalance as indicators for additional medical evaluation; (b) performs a systematic evaluation that includes attention to joint hypermobility [1,77], joint injury [90], skin fragility [6,27], urogenital findings [91], neurologic findings like head/muscle aches or poor balance [52,92], altered immunity with inflammation [14,93,94], and the dysautonomia symptoms of IBS [51], POTS [47,48], and MCAS [49,50]; (c) prioritizes a general EDS–dysautonomia diagnosis before typecasting but looks for unusual findings that would favor severe types [7,8] or other connective tissue dysplasias [64,68]; (d) recognizes that gene panels or genome sequencing are required to screen for the many genes changes being associated with EDS; and (e) emphasizes that most gene mutations will contribute incrementally to a general EDS phenotype rather than to particular EDS types.

This holistic approach could validate as pathophysiologic rather than psychogenic [95] the stress, anxiety, and joint-muscle pain [96] of EDS patients, enable many effective therapies [1,6,7,14,47,77,97,98] before the pain becomes programmed to persist [99], and promote an EDS genetics that matches advances in genomic technology with the advantages of patient-informed experience.

## 5. Conclusions

1. A systematic evaluation of 1261 patients discriminated EDS from less symptomatic hypermobility and showed that autonomic and neurologic findings are integral parts of this disease spectrum.
2. Changes in 317 genes were found by whole exome sequencing analyses of 906 EDS patients and qualified using a novel protocol [14] that emphasizes their relation to finding patterns and clinical mechanisms, rather than to single signs or symptoms.
3. Relevance to EDS of these DNA sequence variants was supported by the presence of 53 mutations in the long-associated collagen type V gene [6], differences from results in 82 developmental disability patients, and previously underemphasized connective tissue laxity symptoms in the diseases associated with these genes (see Table S2).
4. Similar tissue laxity, dysautonomia, and neuromuscular finding profiles were found in 30 EDS groups that averaged 17 patients with changes in the same or related genes; the congruence was interpreted to outline a gene network or entome that can be iteratively disrupted to produce connective tissue dysplasia.
5. The outlined holistic approach for EDS clinical–DNA documentation could shorten diagnostic delays averaging 14 years and promote a sequential correlation of DNA-clinical findings that would fit well with large language artificial intelligence models [79].

## Figures and Tables

**Table 1:** DNA testing of patients with EDS and developmental disability.

**Patients having DNA testing**						
Patients	**EDS (systematic evaluations)**			**Developmental disability (DD)**		
Number	**1261**			**735 ^a^**		
DNA testing (% of patients)	**967 (51)**			461 (63) *		
WES testing (% of patients)	**906 (48)**			112 (15) *		
Significant DNA variant by WES (% of those having WES)	**536 (59)**			**76 (68)**		
Significant DNA sequence variant (% of DNA sequence tests)	**568 (59) ^b^**			**82 (65) ^b^**		
Variant qualified as likely pathogenic or pathogenic by lab	**20 (3.5)**			48 (59) *		
**Qualification and parental origin of relevant DNA variants**						
Categories of DNA variants	**EDS** **All**	**EDS** **Primary**	**EDS** **Additional**	**DD** **All**	**DD** **Primary**	**DD** **Additional**
**Total DNA variants**	**893 ^c^**	**566 ^c^**	**327 ^c^**	**150 ^d^**	**82 ^d^**	**68 ^d^**
VEDU or *VEDUS/O* (%)	384 (43) *	355 (63)	29 (8.9)	53 (35) *	49 (60)	4 (5.9)
VSDU or *VSDUS/O* (%)	324 (36) *	174 (31)	150 (46)	90 (60) *	31 (38)	59 (87)
VMDU or *VMDUS/O* (%)	170 (19)	36 (6.4)	134 (41)	7 (4.7)	2 (2.4)	5 (7.4)
VUDU or *VUDUS/O* (%)	15 (1.7)	1 (0.18)	14 (4.3)	0	0	0
**Nuclear variants**	**735 (82) ^e^**	**473 (84)**	**262 (80)**	**140 (93)**	**80 (98)**	**60 (88)**
maternal origin (%)	246 (33) *	164(35)	82 (31)	29 (21) *	14 (18)	15 (25)
paternal origin (%)	193 (26)	122(26)	71 (27)	41 (29)	15 (19)	26 (43)
De novo (%)	31 (4.2) *	18(3.8)	13 (5.0)	49 (35) *	38 (48)	11 (18)
Unknown (%)	271 (37)	172(36)	99 (38)	21 (15)	13 (16)	8 (13)
**Mitochondrial variants**	**158 (18) ^e^**	**93 (16)**	**65 (20)**	**10**	**2 (2.4)**	**8 (12)**
maternal origin (%)	102 (65)	58 (62)	44 (68)	6 (60)	1 (50)	5 (63)
paternal origin (%)	0	0	0	0	0	0
De novo (%)	4 (2.5)	4 (4.3)	0	0	0	0
Undetermined (%)	52 (33)	31 (33)	21 (32)	4 (40)	1(50)	3 (38)

^a^ All developmental disability (DD) patients had chromosome or DNA testing, 102 of 459 (22%) having copy number variants by microarray, including 11 of the 76 with a significant DNA variant by WES (only 8 of 233 or 3.4% of EDS patients having microarray had copy number variants, 2 encompassing the PMP22 gene qualified as significant in Table S3); ^b^ EDS patients had 31 gene panel tests (18 with variants, all systematically evaluated) and 30 allele tests on 30 EDS relatives of WES-positive patients (19 with variants, 14 systematically evaluated in Table S3), while 6 of 14 DD patients had variants with gene panel tests in Table S4; ^c^ 566 patients had primary DNA variants including 345 (61%) with 1 and 221 (39%) with additional variants totaling 1 in 142 patients (64% of the 221), 2 in 56 patients (25%), 3 in 16 patients (7.2%), 4 in 6 patients (2.7%), and 5 in 1 patient (0.45%); ^cd^ variants judged primary (most relevant to EDS or DD) were all qualified as V*DU (see protocol below Table S3 and text), additional variants as V*DUS/O; ^d^ 82 patients had primary variants including 41 (50%) with 1 and 41 (50%) with additional variants totaling 1 in 22 patients (54% of 41), 2 in 12 patients (29%), 3 in 6 patients (15%), and 4 in 1 patient (2.4%); ^e^ percentages in these rows refer to proportions of all DNA variants; * EDS patient proportions significantly different (*p* < 0.05) from those of DD; “unknown” for nuclear gene variants indicates that no parental samples were analyzed, “undetermined” for mitochondrial DNA variants indicates inability to distinguish de novo or maternal origin.

**Table 2:**
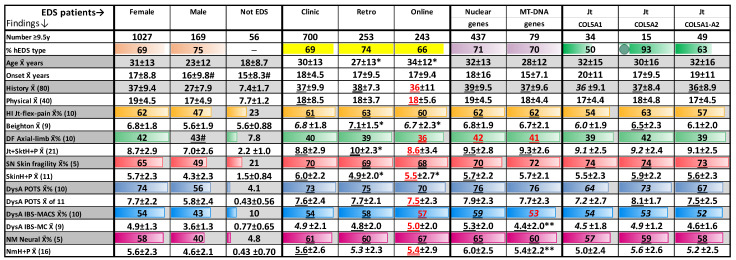
Comparison of category totals and finding frequencies in EDS patient groups.

Four comparisons of EDS patients over 10 (9.5) years are made: (1) females, males and patients not meeting EDS criteria (Not EDS, [46]) with # indicating those few values not significantly different (*p* < 0.05) from females; (2) females (F) with clinic, retrospective (Retro), or online evaluations, values corrected toward the more valid clinic evaluation based on those for females aged 21–40 years (see Methods—increases/decreases in black/red print with italics/underline/double underline for 3–5%/6–10%/11–20% correction, * indicating significant differences from clinic patients); (3) all EDS patients with nuclear versus mitochondrial gene changes, ** indicating significant differences in the latter values; (4) EDS patients with changes in collagen type V alpha-1 or alpha-2 chain genes (*COL5A1*, *COL5A2*) versus the combined groups, values corrected for proportions of male, retrospective, or online patients with black/red print as above, green circle indicating significant difference; category totals (history, physical, Beighton, Jt + SktH + P, etc., are interspersed with finding frequency averages from the mechanism classes of Table S1B: HI (hypermobility) Jt-flex-pain row 61, DF (deformation) axial–limb row 74, SN skin fragility row 80, DysA (dysautonomia) POTS row 94 or IBS-MCAS row 107, and NM neuromuscular row 113.

**Table 3:**
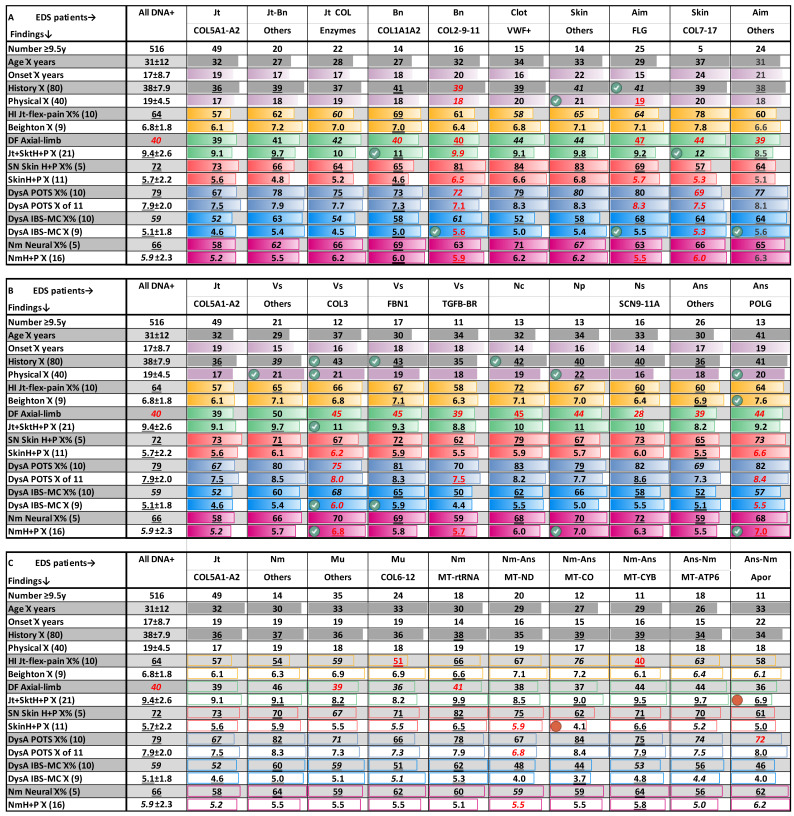
Similar EDS–dysautonomia finding frequencies in patients with recurring gene variants.

Gene abbreviations and patient numbers are detailed in Tables S2–S4, finding category totals and frequency averages of findings classed by mechanism are those of Table S1. Numbers of male, retrospective, or online patients are shown for each group in Table S1B, as are factors correcting for their lower, lower, or higher frequencies relative to clinic female patients (see Section 2). The amount of correction is indicated by italicizing (3–5%), underlining (6–10%), or double underlining (10–20%) the category or frequency number, with black text showing increases and red decreases. The first two columns of the A–C comparisons show the category totals and average frequencies of all 516 patients over age 10 with DNA variants as a statistical reference, then the 49 with well-accepted collagen type V variants [1,6] for easy comparison; 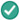
with check, 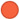
filled, significant difference *p* < 0.05 above, below value for all 516 patients with gene changes; $\bar{X}$, mean; numbers after ± standard deviations.

**Table 4:**
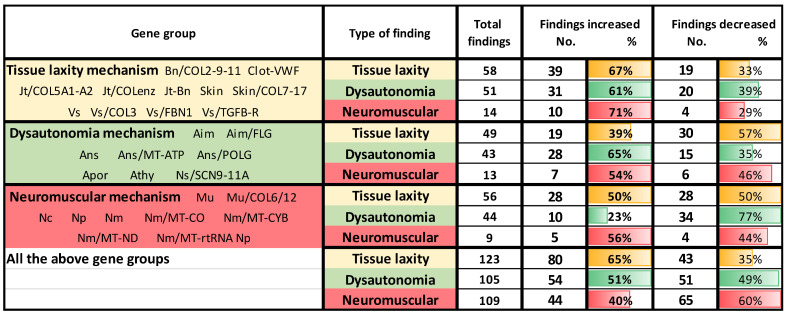
Increases or decreases in EDS finding frequencies correlate with tissue laxity and neuroautonomic mechanism.

Gene group abbreviations in column 1 are explained in Tables S1B or S2, Bn/COL2-9-11 indicating that EDS patients with changes in collagen type II, IX, XI genes having bone impact, etc.; the total number of findings related to tissue laxity, dysautonomia, or neuromuscular mechanisms for all EDS patients having the column 1 gene changes are indicated in columns 2 and 3; the numbers and percentages of these findings that are increased (columns 4 and 5) or decreased (columns 6 and 7) are taken from those under the gene groups in Table S1B, rows 11–47.

## Data Availability

All DNA data on EDS patients will be offered to the ClinGen and Mitomap databases after journal publication, 60–65% of DNA variants have already been entered as shown in Excel Table S3 of the Supplementary Materials. It is hoped that publication after peer review will support the association of these DNA variants with EDS as interpreted by the authors. The databases of EDS and developmental disability DNA variants will be available in the Supplementary Materials as Excel Tables S3 and S4, the EDS1261GW1-23 database as the Excel Tables S5 and S6, matching the positive–negative DNA testing in the latter with the scrambled DNA variants of Table S3 through contact with author G.N.W., as indicated above.

## Supplementary Materials

The following supporting information can be downloaded at https://www.mdpi.com/article/10.3390/cimb46030166/s1, Supplementary Materials include an Excel file (Supplementary Materials for clinical-genomics of EDS Wilson-Tonk.xls) that contains 4 supplemental Tables S1–S4 plus Sheets S5 and S6 containing a deidentified database of 1261 EDS patients from which the components of this article are derived. Patient numbers associated with actual DNA variants in Table S3 are scrambled relative to those in Sheet S6, researchers with qualifications and access to the journal are able to contact the author (GW) for the key to matching numbers.
